# Rational application of targeted therapeutics in mucinous colon/appendix cancers with positive predictive factors

**DOI:** 10.1002/cam4.2847

**Published:** 2020-01-20

**Authors:** Ashokkumar Dilly, Brendon D. Honick, Yong J. Lee, David L. Bartlett, Haroon A. Choudry

**Affiliations:** ^1^ Department of Surgery University of Pittsburgh Medical Center Pittsburgh PA USA

**Keywords:** colonoids, MUC2; mucinous colon/appendix cancer; MEK, PI3K, xenograft

## Abstract

Molecular‐targeted therapies have demonstrated disappointing results against most advanced solid cancers. This may largey be attributed to irrational drug use against unselected cancers. We investigated the efficacy of dual MEK‐PI3K drug therapy against KRAS mutated mucin 2 (MUC2)‐secreting LS174T cells and patient‐derived ex vivo and in vivo models of KRAS mutated mucinous colon/appendix cancers. These tumors demonstrate unique phenotypic and genotypic features that likely predict sensitivity to this targeted co‐therapy. Co‐treatment with MEK inhibitor (trametinib) and PI3K inhibitor (pictilisib)‐induced synergistic cytotoxicity and intrinsic mitochondrial‐mediated apoptosis in LS174T cells and tumor explants in vitro. Dual drug therapy also induced endoplasmic reticulum stress (ERS)‐associated proteins (GRP78/BiP, ATF4, and CHOP). However, CHOP knock‐down assays demonstrated that mitochondrial‐mediated apoptosis in LS174T cells was not ERS‐dependent. Dual drug therapy also significantly decreased MUC2 expression, MUC2 post‐translational modification (palmitoylation) and secretion in LS174T cells, suggesting a simultaneous cytotoxic and mucin suppressive mechanism of action. We also demonstrated effective mucinous tumor growth suppression in ex vivo epithelial organoid (colonoid) cultures and in in vivo intraperitoneal patient‐derived xenograft models derived from mucinous colon/appendix cancer. These promising preclinical data support a role for dual MEK‐PI3K inhibitor therapy in mucinous colon/appendix cancers. We postulate that mucinous KRAS mutated cancers are especially vulnerable to this co‐treatment based on their unique phenotypic and genotypic characteristics.

## Graphical abstract

Mucinous KRAS mutated colon/appendix cancers demonstrate unique genotypic and phenotypic characteristics that allow for the rational application of MEK‐PI3K inhibitor therapy. The present study demonstrates that dual MEK‐PI3K inhibitor therapy (trametinib plus pictilisib) reduces mucinous tumor growth in patient‐derived ex vivo and in vivo models of KRAS mutated mucinous colon/appendix cancer.

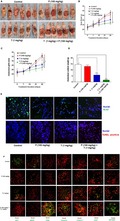

## 1 INTRODUCTION

Tremendous preclinical and clinical researches have been conducted with targeted therapies against mutationally activated oncogenes. Identification of a limited set of “driver” mutations and common downstream oncogenic signaling pathways provides actionable targets for drug therapy.^1^, ^2^, ^3^ Mitogen‐activated protein kinase (MAPK)‐associated RAS‐RAF‐MEK (MAPK kinase/ERK kinase)‐ERK (extracellular signal‐regulated kinase) signaling pathway and phosphatidylinositol‐4,5‐bisphosphate 3‐kinase (PI3K)‐associated AKT‐mTOR (mechanistic target of rapamycin kinase) signaling pathway are commonly altered in solid cancers.^4^, ^5^ Single‐drug MAPK or PI3K pathway inhibitors have demonstrated disappointing results in clinical trials, possibly because cancers depend on multiple oncogenic pathways simultaneously or because of feedback activation of parallel pathways in response to single‐pathway inhibition. Dual‐pathway inhibiting drug combinations have shown promising results in preclinical in vitro and in vivo solid cancer models. Early phase clinical trials testing dual MEK‐PI3K pathway inhibitors in advanced solid cancers have demonstrated safety, although efficacy has been disappointing.^6^, ^7^, ^8^ Major considerations to improve the efficacy of targeted therapies include identification of ideal drugs and drug combinations, optimizing dosing schedules, and enriching for patient factors (eg, phenotype and genotype) that are more likely to respond to specific targeted therapies. However, no clear predictive factors for dual MEK‐PI3K inhibitor therapy have been identified to date.

In this study, we tested the efficacy of dual MEK‐PI3K drug therapy against mucinous colon/appendix cancers since these tumors demonstrate unique phenotypic and genotypic features that are likely to be predictive of sensitivity to this combination therapy. Genotypically, these tumors frequently demonstrate KRAS mutations, resulting in downstream activation of MAPK and PI3K signaling pathways.^9^, ^10^, ^11^, ^12^, ^13^, ^14^, ^15^, ^16^, ^17^, ^18^, ^19^ Phenotypically, mucinous tumors demonstrate high basal endoplasmic reticulum stress (ERS), and associated signaling pathways known as the upregulated protein response (UPR), likely due to high mucin 2 (MUC2) protein turnover.^20^, ^21^, ^22^, ^23^ Therefore, they are likely to be vulnerable to aggravated ERS‐associated apoptosis. However, MAPK and PI3K signaling have been shown to protect some cancers against ERS‐induced cell death through various mechanisms.^24^, ^25^ We hypothesized that dual MEK‐PI3K inhibitor therapy in mucinous colon/appendix cancers would inhibit molecular pathways for survival and sensitize cells to ERS‐induced apoptosis. We also postulated that ERS‐mediated apoptosis would be further enhanced in response to disruption of MUC2 protein homeostasis by PI3K inhibition since PI3K‐AKT‐depedent fatty acid synthase (FASN) activity is essential for appropriate MUC2‐palmitoylation and secretion.^26^ Finally, we postulated that dual cytotoxic and MUC2‐suppressive properties of combined MAPK‐PI3K pathway inhibition would be advantageous in these mucinous tumors.^27^, ^28^, ^29^

In this study, we investigated the efficacy of dual MEK‐PI3K inhibition in established KRAS mutated MUC2‐secreting colon cancer cells (LS174T), and patient‐derived in vitro and in vivo models (tumor explants and epithelial organoid [colonoid] cultures, and intraperitoneal [IP] murine xenografts) derived from KRAS mutated mucinous colon/appendix cancers.

## 2 MATERIALS AND METHODS

### 2.1 Materials

LS174T cells (KRAS p.G12D mutation) and COS‐7 cell lines were obtained from ATCC (Manassas, VA), and Cell line authentication was performed using the Genetica cell line testing a LabCorp brand. Trametinib (MEK inhibitor) and pictilisib (PI3K inhibitor) were purchased from Cayman Chemical. CellTiter 96 Aqueous Assay was obtained from Promega Corporation. Female athymic nude mice were obtained from Jackson Laboratory. Reverse transcription‐polymerase chain reaction (RT‐PCR) kits, including primers and probes for MUC2 and glyceraldehyde 3‐phosphate dehydrogenase (GAPDH), were obtained from Applied Biosystems Inc (ABI). MUC2 antibody for western blot and the in situ 5‐bromo‐2'‐deoxyuridine (BrdU)‐Red DNA Fragmentation kit were obtained from Abcam. MUC2 antibody for immunofluorescence assay was obtained from Santa Cruz Biotechnology. Anti‐rabbit Alexa 647, Alexa 488, and caspase 3 antibodies were obtained from Cell Signaling Technology. Sytox orange for nucleic acid labeling was obtained from Life Technologies. All other chemicals were purchased from Sigma‐Aldrich.

### 2.2 Cell proliferation assay

Briefly, 5000 cells were seeded in a 96‐well culture plate and allowed to adhere overnight. Following treatment, cell viability was determined by adding 3‐(4,5‐dimethylthiazol‐2‐yl)‐5‐(3‐carboxymethoxyphenyl)‐2‐(4‐sulfophenyl)‐2H‐tetrazolium) (MTS) solution and incubating for 2 hours at 37°C. The absorbance of the formazan product at 490 nm was measured directly using an enzyme‐linked immunosorbent assay (ELISA) plate reader.

### 2.3 Annexin V and propidium iodide (PI) staining

LS174T cells were seeded in 60‐mm diameter culture dishes and incubated overnight. Following treatment, Annexin V and PI staining were performed using the FITC Annexin V Apoptosis Detection Kit (BD Biosciences). Data were analyzed by flow cytometry using a BD Accuri c6 instrument and software (BD Biosciences).

### 2.4 Western blot analysis

Cells were lysed using radioimmunoprecipitation assay (RIPA) buffer with protease and phosphatase inhibitors. Ten micrograms of soluble proteins were run on a 4%‐20% gradient sodium dodecyl sulfate‐polyacrylamide gel electrophoresis (SDS‐PAGE) or 1% agarose gel followed by blotting onto nitrocellulose membranes. After blocking the membranes with 5% fat‐free dry milk powder for 60 minutes at room temperature, blots were incubated with specific marker antibodies (all 1:1000) overnight at 4°C. The blots were then washed and incubated with HRP‐conjugated anti‐mouse or anti‐rabbit secondary antibodies for 60 minutes at room temperature. After washing, blots were developed with enhanced Super Signal West Dura Extended Duration Substrate and visualized using a Bio‐Rad imager with chemiluminescence capability.

### 2.5 Stable cell line generation

LS174T cells were incubated with CCAAT‐enhancer‐binding protein homologous protein (CHOP) or PUMA (p53‐upregulated modulator of apoptosis) short‐hairpin RNA (shRNA, h) lentiviral particles and 5 µg/mL final concentration polybrene (Sigma‐Aldrich, #9268). Following 24 hours of incubation, the medium was replaced with complete DMEM for 24 hours and then puromycin was added at a final concentration of 3 µg/mL. Cell were sub‐cultured for 3 weeks under puromycin selection to eliminate non‐transduced cells.

COS‐7 cells were transfected with pSNMUC2‐MG vector expressing MUC2 N‐terminal (a gift from G. Hansson, University of Gothenburg, and Gothenburg, Sweden). Following overnight incubation, the medium was replaced with complete DMEM for 24 hours and then G418 was added at a final concentration of five hundred microgram per ml to eliminate non‐transfected cells.

### 2.6 Real‐time PCR

Total RNA was isolated from LS174T cells using Qiagen RNA isolation kit. The cDNA was prepared using the Quanta cDNA synthesis kit. Real‐time PCR was then carried out using an ABI Prism SDS 7000 Cycler system, using commercially available primers and probe obtained from ABI for specific cDNA, for 40 cycles at 95°C for 15 seconds. All PCR reactions were performed in triplicate, the house keeping gene GAPDH was used as a reference gene for the mRNA levels of genes of interest.

### 2.7 Immunofluorescence

Tumor tissues were embedded in OCT medium‐containing cryomolds and immediately frozen in 2‐methyl‐butane. Then, 5 µm frozen tissue sections were cut using a cryostat and layered on super frost plus slides, which were fixed in 4% para‐formaldehyde for 15 minutes, washed, and blocked for 60 minutes at room temperature. The slides were then stained for 3 hours at room temperature with specific antibodies. The slides were washed five times with 1 × phosphate‐buffered saline (PBS) and incubated with secondary anti‐rabbit Alexa 488 and anti‐mouse Alexa 647 antibodies, and nuclear dye SYTOX Orange for 30 minutes at room temperature. The slides were washed five times with 1 × PBS. Cover slips were mounted on the sections using ProLong Gold Anti‐fade solution. The stained slides were examined using a confocal microscope (Leica TCS SL DMRE microsystems).

### 2.8 Terminal deoxynucleotidyl transferase dUTP nick end labeling (TUNEL) assay

In situ BrdU‐Red DNA Fragmentation kit was used to detect apoptosis in frozen tissues. Br‐dUTP staining was used to detect the DNA strand breaks. Briefly, frozen sections were deparaffinized, permeabilized using proteinase K, DNA strand breaks were end‐labeled with terminal transferase, and then visualized using fluorescence microscopy.

### 2.9 Colonic crypt isolation and culture

Fresh primary KRAS mutated colon/appendix mucinous neoplastic tissue (5 tumors with KRAS p.G12D mutations and 3 with KRAS p.G12V mutations) was used to develop ex vivo epithelial organoid cultures (colonoids) based on a previously published protocol.^30^ Human tissue was collected under an Institutional Review Board (IRB)‐approved protocol, and informed consents were obtained from all patients prior to analysis. The mucosa was stripped of the underlying muscle layer and tumor tissue fragments were washed, followed by incubation in chelation solution supplemented with ethylene diamine tetraacetic acid (EDTA, 2 mmol/L final concentration). Basal culture medium (advanced DMEM/F12 supplemented with penicillin/streptomycin, 10 mmol/L HEPES, and Glutamax) was added, and the crypts were washed twice with basal culture medium and suspended in Matrigel matrix (MM). The MM was polymerized by incubation at 37°C in a 5% CO_2_ incubator for 30 minutes and then overlaid with human intestinal stem cell medium.

### 2.10 Tumor explant culture

Tissue from fresh primary KRAS mutated colon/appendix mucinous tumors (5 tumors with KRAS p.G12D mutations and 3 with KRAS p.G12V mutations) was obtained during surgery. The explant culture system was used according to a previously described method.^31^ Using a 4‐mm biopsy puncher, cubes of tumor tissue were prepared and placed in antibiotic gentamicin containing DMEM and 10% FBS (typically three cubes/well in 24‐well plates). The tumor explants were cultured at 37°C in a humidified atmosphere containing 5% CO_2._

### 2.11 Alcian blue stain

The VitroView^Tm^ Alcian Blue Stain kit (GeneCopoeia Rockville, MD) was used to stain mucin in frozen tissues.

### 2.12 JC‐1 staining for mitochondrial membrane potential

The JC‐1 Mitochondrial Membrane Potential Assay kit, (Cayman Chemical) was used to monitor mitochondrial transmembrane potential (ΔΨm). Cells were stained with 100 µL/mL JC‐1 staining solution in culture medium in a 24‐well plate and incubate at 37°C for 15 minutes. In the undamaged mitochondria, the aggregated dye induces red fluorescence, whereas in apoptotic cells with altered ΔΨm, the dye remains as monomers in the cytoplasm with diffuse green fluorescence.

## 3 MUC2 ELISA

Conditioned medium from cell/ COS‐7 cells expressing MUC2 N‐terminal (50 µL/well) was coated onto a Corning 96‐well EIA/RIA plate by incubating overnight at room temperature in 0.1 mol/L carbonate buffer pH 9.6. Plates were blocked for 1 hour at room temperature with 2% bovine serum albumin (BSA) in PBS and incubated overnight with MUC2 antibody in PBS containing 0.05% Tween‐20. Bound MUC2 antibody was detected using anti‐mouse HRP‐conjugated and 2,2'‐azino‐bis (3‐ethylbenzothiazoline‐6‐sulphonic acid (ABTS) substrate (Sigma‐Aldrich St. Louis, MO).

### 3.1 In vitro acyl‐biotin exchange (ABE) assay

The ABE assay used was adapted from Wan and colleagues with modifications..^32^ COS‐7 Cells were lysed in lysis buffer (LB, pH 7.4) containing 50 mmol/L Tris‐HCl pH 7.4, 150 mmol/L NaCl, 1% NP‐40, 1 mmol/L EDTA, and protease inhibitors. For this procedure, all centrifugation steps were carried out at 850 × *g* for 5 minutes. MUC2 was first immunoprecipitated from 500 µg protein using 6 µg anti‐MUC2 antibody, and the MUC2 and anti‐MUC2 antibody complexes were bound to the exosome immunoprecipitation reagent (Protein G, #10612D, Fisher scientific). Then, 50 mmol/L of N‐ethylmaleimide (NEM, E3876, Sigma‐Aldrich) in LB, pH 7.4, was added to the immunoprecipitated MUC2 and incubated for 3 hours at 4°C with gentle rotation to block free thiols of cysteine residues. After three washes with LB, pH 7.4, MUC2 was treated with and without (mock as control) 1 mol/L hydroxylamine (HAM, #379921, Sigma‐Aldrich) in LB, pH 7.4 for 2 hours at room temperature with gentle rotation. MUC2 was then rinsed three times with LB, pH 6.2 followed by treatment with 5 µmol BMCC‐Biotin (#21900, Thermo Fischer Scientific) in LB (pH 6.2) overnight at 4°C with gentle rotation. This was followed by three rinses with LB (pH 7.4) to remove excess biotin, and MUC2 was then eluted with reducing sample buffer. Samples were analyzed using SDS‐PAGE.

### 3.2 Patient‐derived xenograft (PDX) model

A previously developed IP murine PDX model of KRAS mutated (KRAS p.G12D mutation) mucinous appendix cancer has been published.^27^ Murine experiments were conducted under an Institutional Animal Care and Use Committee (IACUC)‐approved protocol. We calculated sample size for the animal experiments using the following formula, corrected sample size = sample size/(1 − [% attrition/100]); we expected a 20% attrition rate in our PMP‐PDX model.^33^ Animals were randomized on day 7 after tumor inoculation to different treatment groups (eight animals per group) and weekly measurements of gross body weight (g) and abdominal girth (mm) were recorded. All animals were euthanized at the same time once IACUC criteria were reached for any of the mice (maximum abdominal girth of 30 mm, inanition, respiratory compromise, evidence of pain, >20% reduction in body weight, scruffy appearance), at which point the abdominal contents (organs plus mucinous tumor) were harvested en bloc and weighed.

### 3.3 Statistical analysis

GraphPad Prism 5 software (GraphPad Software) was used for statistical analysis. Two‐group comparisons were performed using the Student *t* test. Comparisons among more than two groups were assessed using an analysis of variance (ANOVA) with post hoc testing.

## 4 RESULTS

### 4.1 Combination of trametinib and pictilisib induced synergistic cytotoxicity and apoptosis in vitro

**Figure 1:**
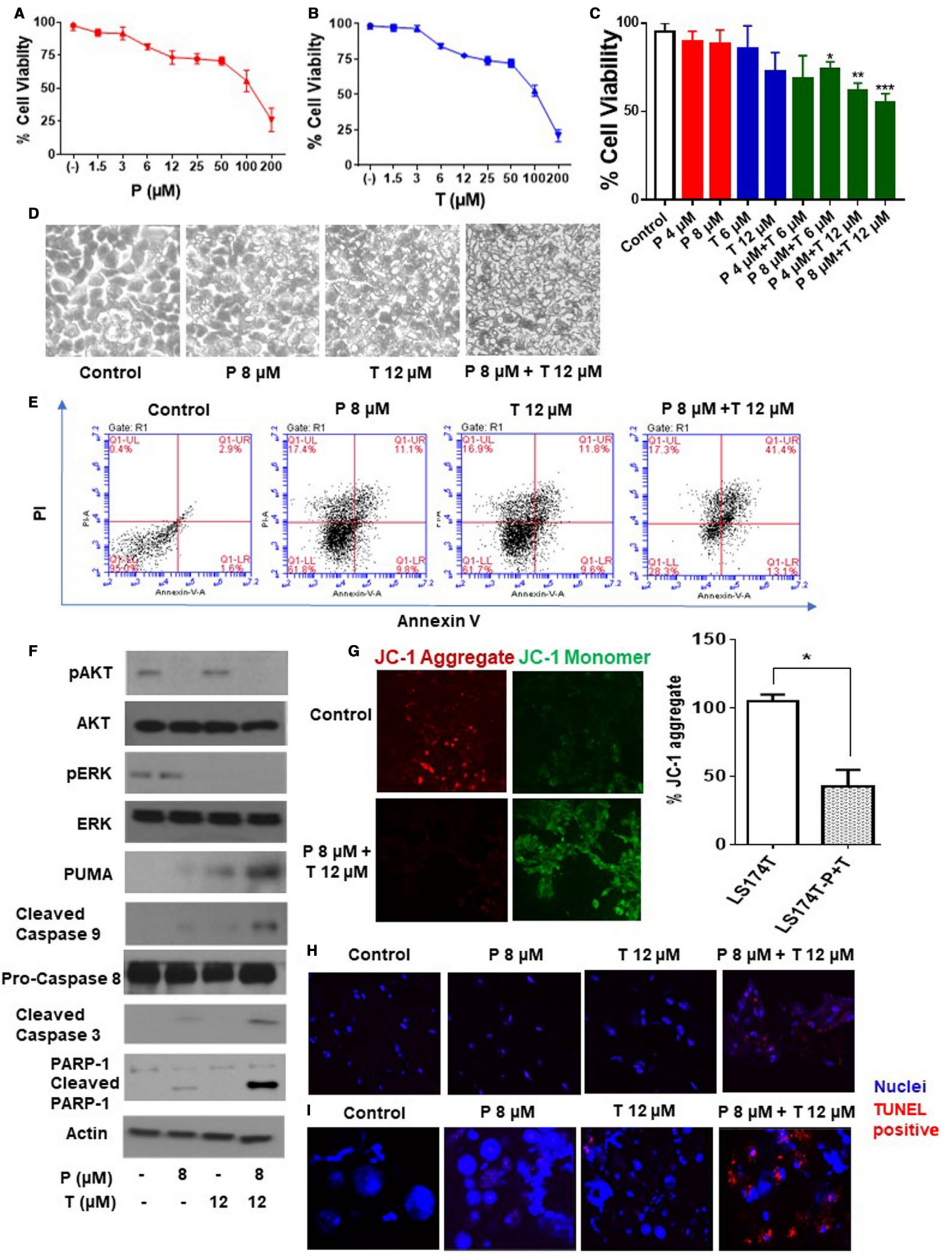
Combination of pictilicsib and trametinib induced synergistic cytotoxicity and apoptosis in vitro. LS174T cells were treated with trametinib (T) alone (0‐200 µmol/L) (A), pictilisib (P) alone (0‐200 µmol/L) (B), or combination of P (0‐8 µmol/L) and T (0‐12 µmol/L) for 24 h (C), following which cell viability was analyzed by MTS assay. Combination index (CI) for combination treatment (P 8 µmol/L + T 12 µmol/L) in (C) was calculated using computer software Compusyn. LS174T cells were treated with single or dual drug therapy for 24 h following which apoptotic cells were analyzed by phase contrast microscope (D) and flow cytometry (Annexin V/PI staining) (E) at 24 h. Following treatment of LS174T cells with single or dual drugs, signaling pathway molecules (AKT, ERK) and apoptotic marker proteins (caspase 3/8/9, PARP‐1, and PUMA) were measured using western blot assay (F). LS174T cells were treated with combination of P 8 µmol/L + T12 µmol/L for 24 h and stained with JC‐1. Diffuse green JC1‐monomers indicate mitochondrial depolarization (damage), and punctate red JC1‐aggregates indicate intact mitochondrial membrane potential (ΔΨm). Percentage (%) JC‐1 aggregate staining was quantified. Confocal images were randomly taken of 10 different fields and analyzed using Image J Software to quantify the average intensity of the protein expression (G). Human tumor explant cultures (H) and colonoid cultures (I) were treated with single or dual drug therapy for 24 h and apoptosis was measured by TUNEL assay. Blue color represents nuclei and red color represents TUNEL positive cells. Error bars represents standard deviation (SD) from triplicate experiments (*P < .05, **P < .01, ***P < .001)

Treatment of LS174T cells with varying doses of trametinib or pictilisib (0‐200 µmol/L) for 24 hours resulted in dose‐dependent decrease in cell viability, measured by MTS assay (Figure 1A,B). We demonstrated that the IC50 dose for trametinib was 117µM and for pictilisib was 120 µmol/L. Dual drug therapy (trametinib + pictilisib) induced synergistic cell death, with approximately 50% decrease in cell viability following exposure to trametinib (12 µmol/L) plus pictilisib (8 µmol/L) for 24 hours (combination index calculated using the computer software Compusyn was 0.2) (Figure 1C,D). Combination therapy (trametinib + pictilisib) was more effective than single drug therapy at inducing apoptotic cell death, as demonstrated by dual AnnexinV/PI staining at 24 hours (Figure 1E). As expected, dual drug treatment reduced phosphorylated‐ERK and ‐AKT protein levels (consistent with MAPK/PI3K signaling inhibition). At the same time, protein levels for PUMA, cleaved caspases 9/3 (but not caspase 8), and cleaved poly‐ADP ribose polymerase‐1 (PARP‐1) increased following dual drug therapy (consistent with mitochondrial‐mediated apoptotic cell death) (Figure 1F). Similar changes in MAPK/PI3K signaling proteins (phosphorylated‐ERK and ‐AKT) and apoptotic proteins (PUMA, cleaved caspases 3/9, PARP‐1) following drug therapy were confirmed in explant tissue from mucinous colon/appendix cancers (Figure S1). We assessed changes in mitochondrial transmembrane potential (ΔΨm) following dual drug therapy using mitochondrial membrane‐permeant fluorescence dye JC‐1. JC‐1 aggregates (reduced red‐fluorescent J‐aggregates) were significantly reduced following co‐treatment, consistent with mitochondrial membrane damage and activation of intrinsic apoptosis (Figure 1G). These data suggest that dual drug therapy (trametinib + pictilisib) induced synergistic cell death via the intrinsic mitochondrial‐mediated apoptotic pathway in LS174T cells. We confirmed the therapeutic efficacy of this combination treatment using explant tissue (Figure 1H) and colonoid cultures (Figure 1I) derived from primary KRAS mutated mucinous colon/appendix cancers. Using TUNEL assay, we found a pronounced increase in apoptotic cells following dual drug therapy for 24 hours.

### 4.2 Dual drug therapy‐induced apoptosis was independent of ERS aggravation in vitro

**Figure 2:**
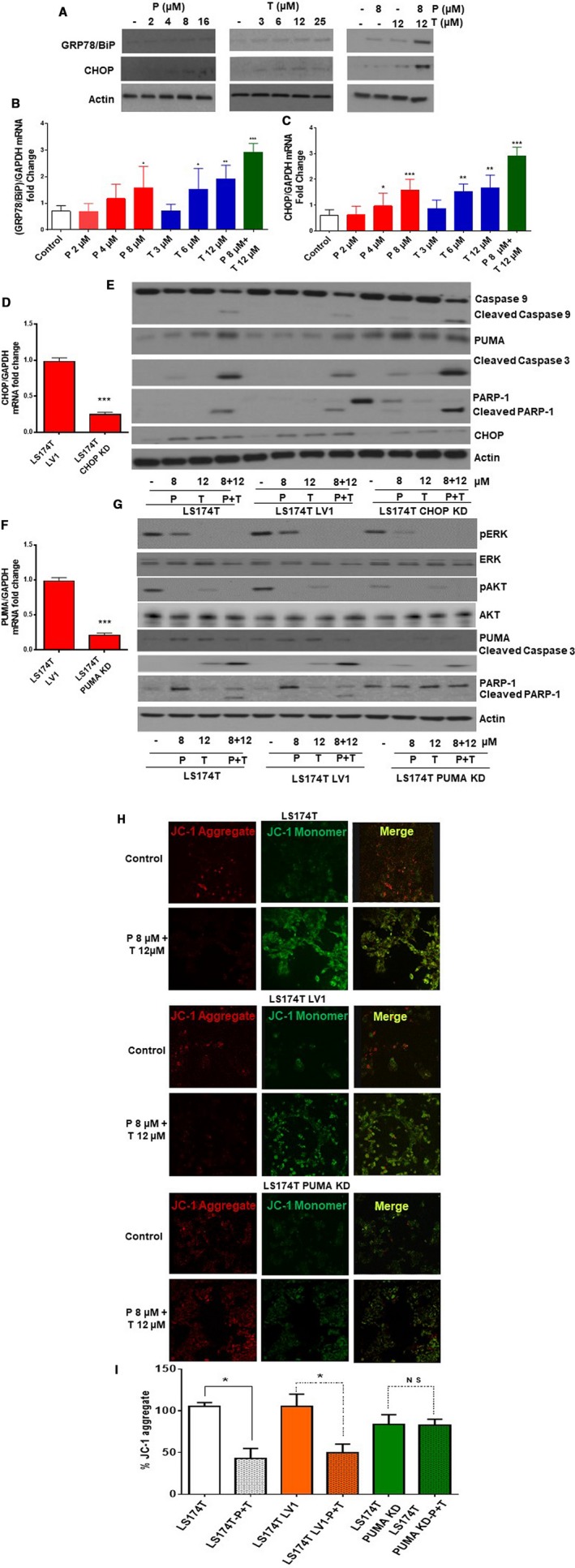
Dual drug therapy‐induced apoptosis was independent of ERS aggravation in vitro. LS174T cells were treated with pictilisib (P) alone, trametinib (T) alone, or combination of P + T for 24 h following which changes in ERS markers (GRP78/BiP, CHOP) were measured using western blot assay (A) and qRT‐PCR (B‐C). LS174T cells were transfected with CHOP (D) or PUMA (F) shRNA (h) Lentiviral Particles to generate stable CHOP or PUMA knockdown (KD) LS174T cells respectively; knockdown efficiency was determined by qRT‐PCR. LS174T, LS174T Lentiviral control (LV1), and LS174T CHOP KD cells were treated with P alone, T alone or combination (P + T) for 24 h and lysates were probed with CHOP, Caspase 3/9, PUMA, and PARP‐1 antibodies (E). LS174T, LS174T Lentiviral control (LV1), and LS174T PUMA KD cells were treated with P alone, T alone or (P + T) for 24 h and lysates were probed with pAKT, AKT, pERK, ERK, PUMA, Caspase 3, and PARP‐1 antibodies (G). LS174T cells, LV1, and LS174T PUMA KD cells were treated with combination of P 8 µmol/L + T12 µmol/L for 24 h and stained with JC‐1. Diffuse green JC1‐monomers indicate mitochondrial depolarization (damage), and punctate red JC1‐aggregates indicates intact mitochondrial membrane potential (ΔΨm). Percentage (%) JC‐1 aggregate staining was quantified. Confocal images were randomly taken of 10 different fields and analyzed using Image J Software to quantify the average intensity of the protein expression (H‐I). Error bars represent standard deviation (SD) from triplicate experiments (*P < .05, **P < .01, ***P < .001)

Explant tissues derived from KRAS mutated mucinous colon/appendix cancers demonstrate higher basal ERS‐associated UPR marker levels compared to their non‐mucinous counterparts (Figure S2). We therefore postulated that mucinous tumors may be more susceptible to drug therapies that aggravate ERS. We found that LS174T cells co‐treated with trametinib (12 µmol/L) and pictilisib (8 µmol/L) for 24 hours demonstrated significantly higher levels of UPR proteins, including glucose‐regulated protein 78 kDa (GRP78)/binding immunoglobulin protein (BiP), and CHOP, suggesting ERS aggravation (Figure 2A‐C). Similar changes in ERS protein levels following drug therapy were confirmed in explant tissue from KRAS mutated mucinous colon/appendix cancers (Figure S3). Stable knockdown (KD) of CHOP in LS174T cells (Figure 2D) did not influence the induction of intrinsic apoptosis by dual drug therapy, as demonstrated by persistent activation of PUMA, and cleavage of caspase 9/3 and PARP‐1 in LS174T CHOP KD cells (Figure 2E). Conversely, stable KD of PUMA in LS174T cells (Figure 2F) significantly reduced intrinsic apoptosis following dual drug therapy for 24 hours, suggesting PUMA mediated apoptosis (Figure 2G). This was confirmed by the lack of ΔΨm change (persistent red‐fluorescent J‐aggregates) following dual drug therapy in PUMA KD LS174T cells (Figure 2H‐I). These data suggest that dual drug therapy aggravated ERS in LS174T cells and induced PUMA‐mediated intrinsic apoptosis, however drug‐mediated apoptosis was independent of ERS aggravation.

### 4.3 Dual drug therapy reduced MUC2 expression and secretion in vitro

**Figure 3:**
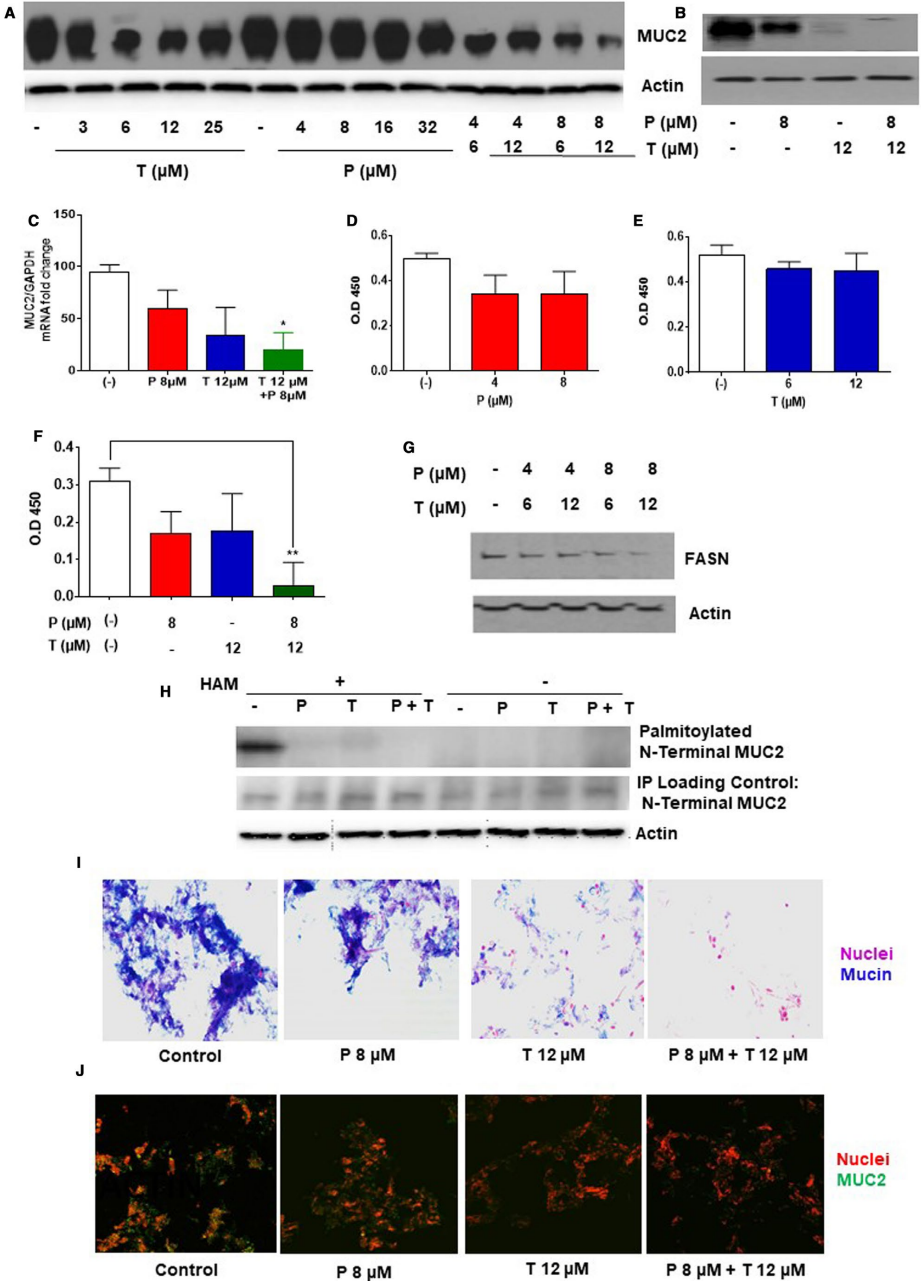
Dual drug therapy reduced MUC2 expression and secretion in vitro*:* LS174T cells (A) and mucinous tumor explants (B) were treated with trametinib (T) alone or pictilicsib (P) alone or combination treatment (P + T) for 24 h. MUC2 protein expression was quantified using western blot assay. Following drug therapy in LS174T cells for 24 h, MUC2 mRNA expression was quantified using qRT‐PCR (C). Conditioned medium was collected following 24 h of single or dual drug treatment and secreted MUC2 protein level was quantified using ELISA (D‐F). Fatty acid synthase (FASN) expression level was quantified by western blot assay following dual drug therapy for 24 h (G). N‐terminal MUC2‐palmitoylation in COS‐7 cells stably expressing MUC2 N‐terminal following single or dual drug therapy for 24 h was determined by ABE assay and quantified by western blot assay (H). Hydroxylamine (HAM), a strong reducing agent that cleaves palmitate from cysteine residues, is necessary for biotinylation. The omission of HAM cleavage (HAM ‐) serves as negative control. Human tumor colonoid cultures were stained with alcian blue (mucin stains blue) (I) or MUC2 antibody (green MUC2 IF) (J) following single or dual drug therapy for 24 h. Error bars represents standard deviation (SD) from triplicate experiments (*P < .05, **P < .01)

Treatment of LS174T cells with trametinib resulted in a dose‐dependent decrease in MUC2 protein expression at 24 hours. This effect was more pronounced following dual drug therapy with trametinib and pictilisib. While dual drug therapy reduced cell viability to approximately 50% in the MTS assay (Figure 1C), MUC2 protein expression was reduced by more than 90% (Figure 3A). We confirmed the MUC2 protein‐suppressive effect of dual drug therapy in explant tissue from KRAS mutated mucinous colon/appendix cancers, demonstrating reduced MUC2 protein (Figure 3B) and mRNA (Figure 3C) expression levels at 24 hours.

We also assessed the effect of dual drug therapy on MUC2 secretion in LS174T cells. MUC2 protein levels were not significantly reduced in conditioned media following treatment with pictilisib or trametinib alone, however dual drug therapy was associated with a pronounced reduction in MUC2 secretion at 24 hours (Figure 3D‐F). Dual drug therapy significantly reduced FASN expression, an enzyme necessary for MUC2 N‐terminal palmitoylation and secretion (Figure 3G). In order to study the effect of dual drug therapy on MUC2 post‐translation modification, we performed ABE assay in COS‐7 cells stably expressing MUC2 N‐terminal. Our data demonstrate a significant reduction in MUC2 N‐terminal palmitoylation following dual drug therapy, that would lead to a reduction in protein secretion (Figure 3H). The overall inhibitory effects of drug therapy on mucin/MUC2 production was confirmed in colonoid cultures from KRAS mutated colon/appendix cancers. Dual drug therapy resulted in a significant reduction mucin (alcian blue) and MUC2 protein (MUC2 antibody) staining at 24 hours (Figure 3I,J).

### 4.4 Combination of trametinib and pictilisib reduced mucinous tumor growth in vivo

**Figure 4:**
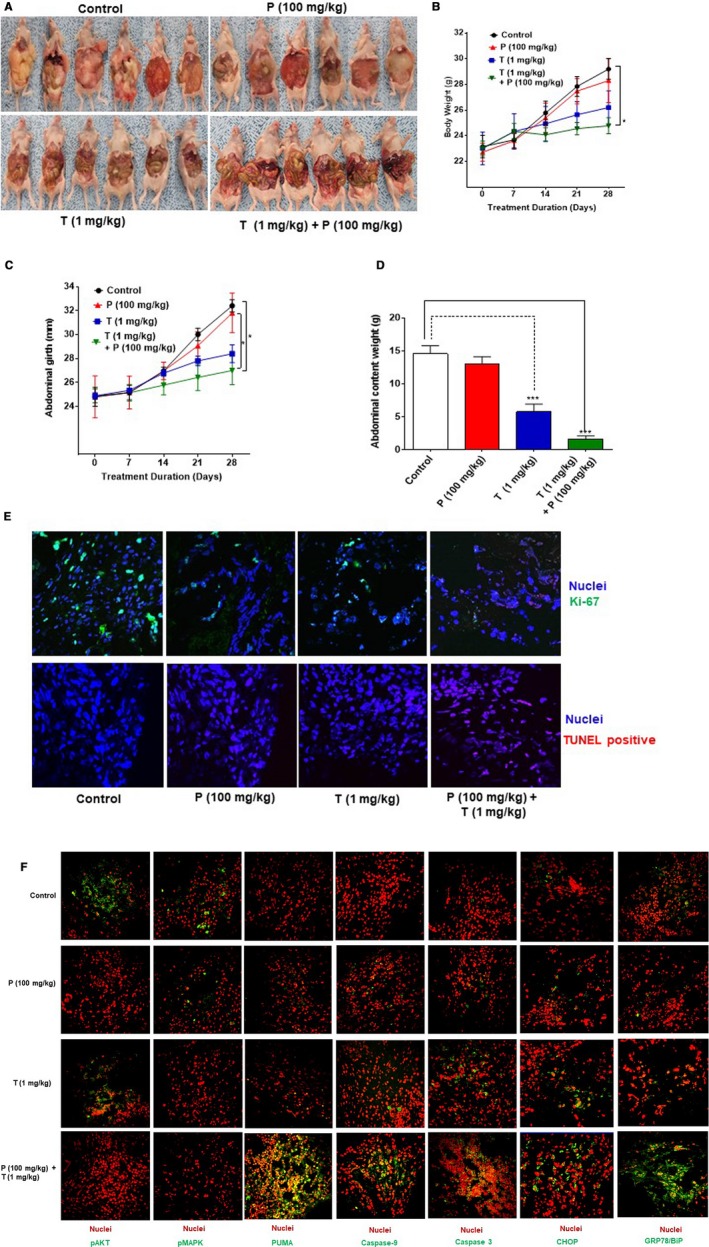
Dual drug therapy reduced mucinous tumor growth in vivo: Patient‐derived xenografts were treated with PBS alone (control), or pictilicsib (P) alone (100 mg/kg b.w.), or trametinib (T) alone (1 mg/kg b.w.), or combination of P + T every other day (starting on day 7 following tumor implantation) until they were euthanized at day 28. Gross intra‐abdominal tumor burden is depicted pictorially (A). Weekly measurements of body weight (g) (B) and abdominal girth (mm) (C) were recorded. At the time of sacrifice abdominal contents weight (tumor + organs, in grams) was quantified (D). Harvested tumor tissue from euthanized mice was stained with proliferation marker (Ki67 IF assay) antibody and TUNEL assay was also performed (E). IF assay was performed for signaling pathway proteins (pAKT and pMAPK), ERS protein levels (GRP78/BiP and CHOP) and apoptosis proteins (Caspase 3/9 and PUMA) following 3 weeks of drug therapy (F). Error bars represent standard error of the mean among the 6 xenograft specimens. Asterisk represents a statistically significant difference compared with the control group (*P < .05, ***P < .001)

The above data suggest a dual cytotoxic and MUC2‐inhibitory effect of combination treatment with trametinib and pictilisib in mucinous colon/appendix cancers. We evaluated the therapeutic efficacy of this combination in vivo using IP PDX models derived from KRAS mutated mucinous appendix cancer. Seven days following IP tumor inoculation, animals were treated IP with vehicle (PBS), trametinib alone (1 mg/kg), pictilisib alone (100 mg/kg) or combination of trametinib and pictilisib, every other day for 3 weeks. Drug dose selection for IP trametinib and pictilisib was based on prior publications and our pilot studies.^34^, ^35^ Treatment‐related drug toxicity was not encountered in the in vivo experiments. Dual drug therapy resulted in significant reduction in mucinous tumor growth, compared to either drug alone, as demonstrated by clinical assessment, serial anthropometric measurements (body weight and abdominal girth), and total abdominal contents at the time of euthanasia (Figure 4A‐D). Tumor tissue harvested from euthanized animals following 3 weeks of therapy demonstrated a significant reduction in cell proliferation (Ki67 staining) and increase in apoptosis (TUNEL positive staining) with dual therapy (Figure 4E). Changes in signaling pathway proteins (pAKT and pMAPK), ERS protein levels (GRP78/BiP and CHOP) and apoptosis proteins (Caspase 3/9 and PUMA) were consistent with those seen in in vitro studies (Figure 4F).

## 5 DISCUSSION

Early phase clinical trials of dual MEK‐PI3K inhibitor therapy for advanced solid cancers demonstrate low response rates but acceptable toxicity.^6^, ^8^ Response rates and disease control rates are low in unselected patients; approximately 5% and 19%, respectively.^6^, ^8^ No clear predictive factors have been identified, although response rates appear to be higher for RAS‐ or RAF‐mutated cancers than for those without these mutations. A phase 1 study (NCT00996892) tested co‐therapy with MEK (cobimetinib) and PI3K (pictilisib) inhibitors against unselected solid cancers. Partial responses occurred in 3 of 78 patients (importantly, all 3 had KRAS or BRAF mutations) and stable disease for more than 5 months was seen in 5 patients.^36^ Another phase 1 trial (NCT01392521) involving MEK (refametinib) and PI3K (copanlisib) inhibitors, refametinib in advanced solid cancers demonstrated a single patient with partial response and 9 with stable disease out of 49 enrolled patients.^37^ A phase 1 study of MEK (trametinib) and PI3K (buparlisib) inhibitors was conducted in 113 patients with RAS or RAF mutant non‐small cell lung, ovarian, and pancreatic cancers. Promising antitumor activity against KRAS (especially G12V) mutant ovarian cancers was seen, including a response rate of 29% (one complete and five partial responses), disease control rate of 76%, and median progression‐free survival of 7 months. However, significant toxicity was encountered with treatment‐related grades 3/4 adverse events documented in 65% of patients.^38^ A phase 1 study of MEK (binimetinib) and PI3Kα (alpelisib) inhibitors was conducted in 58 patients with RAS or RAF mutant advanced solid cancers. Partial responses were documented in 3 of 4 patients with KRAS mutant ovarian cancers.^39^ These data suggest that dual MEK‐PI3K therapy is likely to be effective against RAS mutant cancers.

Mucinous colon/appendix cancers display phenotypic and genotypic characteristics that are predictive of responses to dual MEK‐PI3K inhibition. Mucinous colon cancers are more likely to occur in younger female patients; particularly the right colon; follow the serrated pathway for carcinogenesis; and are more likely to have RAS, RAF, and GNAS mutations.^11^, ^17^, ^40^, ^41^, ^42^ Similarly, mucinous appendix cancers are almost always KRAS mutant, and low‐grade histology almost uniformly demonstrates GNAS mutations.^9^, ^12^, ^13^, ^14^, ^15^, ^16^, ^18^ Identification of novel effective therapies for KRAS mutant colon/appendix cancers is important since KRAS mutation is associated with dysregulated growth, resistance to anti‐EGFR (epithelial growth factor receptor) therapy, and worse survival.^43^ Based on the unique phenotypic and genotypic characteristics of mucinous colon/appendix cancers, we utilized a novel combination of MEK (trametinib) and PI3K (pictilisib) inhibitors in this study. We demonstrated synergistic cytotoxicity, aggravation of ERS and activation of intrinsic mitochondrial‐mediated apoptosis following combination therapy. These findings were consistent across a variety of experimental models, including an established KRAS mutated MUC2‐secreting colon cancer cell line (LS174T), as well as patient‐derived tumor explants, colonoid cultures, and murine IP xenografts of KRAS mutated mucinous colon/appendix cancers. Apoptosis following drug therapy was PUMA‐mediated but ERS‐independent, despite ERS aggravation after dual drug therapy. Importantly, we demonstrated promising preclinical data of mucinous tumor growth suppression in ex vivo colonoid cultures and in vivo IP PDX models derived from KRAS mutant colon/appendix cancers. Furthermore, synergistic interaction between trametinib and pictilisib effectively suppressed tumor growth at lower (less toxic) doses of each individual drug. Importantly, no treatment‐related drug toxicity was encountered in the in vivo experiments.

A unique aspect of mucinous colon/appendix cancers is the abundant production and secretion of MUC2 protein. Although the role of ubiquitously expressed MUC2 in these tumors remains unclear, tumors are generally known to aberrantly express mucins to promote cell survival.^44^ Extracellular mucus may provide a protective barrier around neoplastic cells to shield them against external insults including systemic chemotherapy. In fact, mucinous colon/appendix cancers are more chemo‐resistance than their non‐mucinous counterparts. Therefore, targeting mucin production and secretion may be a viable therapeutic strategy in these tumors, as we have previously demonstrated.^27^, ^28^, ^29^, ^45^ RAS pathway activation has been shown to regulate transcriptional control of mucin production.^46^ We previously demonstrated that MEK inhibition decreased MUC2 expression via downregulation of phosphorylated‐ERK1/2, NF‐κB p65 protein signaling, and reduced AP1 transcription factor binding to the MUC2 promoter.^29^ PI3K signaling plays a prominent role in FASN‐mediated MUC2 palmitoylation, an essential step for normal MUC2 secretion.^26^ In this study, we demonstrated effective reduction in MUC2 expression and secretion following dual MEK‐PI3K inhibition. Dysregulated post‐translational modification and secretion of proteins can induce severe and persistent ERS that can trigger molecular pathways associated with cell death through the activation of UPR molecular signaling pathways. Mucinous tumors demonstrate higher basal ERS, which makes them more vulnerable to ERS aggravation‐induced apoptosis.^20^, ^21^, ^22^, ^23^ Conversely, elevated MAPK and PI3K signaling has been shown to protect cancer cells from ERS‐mediated apoptosis by providing a mechanism for escape.^24^ Our data demonstrate significant induction of ERS response proteins following dual drug therapy. While intrinsic mitochondrial‐mediated apoptosis was increased following co‐therapy, this was not ERS‐dependent.

The research data presented in this study have a number of important limitations. We did not include multiple cell lines in this study and instead utilized patient‐derived explant tissue, colonoid cultures and xenografts derived from mucinous colon/appendix cancers to assess therapeutic effect and corroborate cell line data. We feel that these clinically relevant models are especially important for mucinous tumors for which availability of high‐mucin (MUC2) secreting cell lines is limited. The mechanism for apoptosis induction was not clearly defined in this study and in fact our hypothesis for ERS aggravation playing a dominant role was not borne out in our experimental studies. While ERS was increased following dual MEK‐PI3K therapy, the degree of ERS aggravation may not have been sufficient to drive apoptosis and/or MAPK/PI3K‐mediated escape mechanisms may have protected cancer cells from ERS‐mediated apoptosis. Pictilisib treatment alone was minimally effective at reducing mucinous tumor growth in vivo, likely due to the dominant role of MAPK signaling in these mucinous tumors and crosstalk between PI3K and MAPK cancer signaling pathways.

In summary, we provide promising preclinical data to support the use of dual MEK‐PI3K inhibitor therapy in patients with mucinous colon/appendix cancers. We postulate that these highly mucinous KRAS mutated cancers are especially vulnerable to this co‐treatment based on their phenotypic and genotypic characteristics.

## Data Availability

The data that support the findings of this study are available from the corresponding author upon reasonable request.

## References

[1] Baudino TA . Targeted cancer therapy: the next generation of cancer treatment. Curr Drug Discov Technol. 2015;12(1):3‐20. 26033233 10.2174/1570163812666150602144310

[2] Huang M , Shen A , Ding J , Geng M . Molecularly targeted cancer therapy: some lessons from the past decade. Trends Pharmacol Sci. 2014;35(1):41‐50. 24361003 10.1016/j.tips.2013.11.004

[3] Vogelstein B , Papadopoulos N , Velculescu VE , Zhou S , Diaz LA Jr , Kinzler KW . Cancer genome landscapes. Science. 2013;339(6127):1546‐1558. 23539594 10.1126/science.1235122 PMC3749880

[4] Burotto M , Chiou VL , Lee JM , Kohn EC . The MAPK pathway across different malignancies: a new perspective. Cancer. 2014;120(22):3446‐3456. 24948110 10.1002/cncr.28864 PMC4221543

[5] Courtney KD , Corcoran RB , Engelman JA . The PI3K pathway as drug target in human cancer. J Clin Oncol. 2010;28(6):1075‐1083. 20085938 10.1200/JCO.2009.25.3641 PMC2834432

[6] Jokinen E , Koivunen JP . MEK and PI3K inhibition in solid tumors: rationale and evidence to date. Ther Adv Med Oncol. 2015;7(3):170‐180. 26673580 10.1177/1758834015571111 PMC4406912

[7] Jokinen E , Laurila N , Koivunen JP . Alternative dosing of dual PI3K and MEK inhibition in cancer therapy. BMC Cancer. 2012;12:612. 23259591 10.1186/1471-2407-12-612 PMC3563486

[8] Tolcher AW , Peng W , Calvo E . Rational approaches for combination therapy strategies targeting the MAP kinase pathway in solid tumors. Mol Cancer Ther. 2018;17(1):3‐16. 29295962 10.1158/1535-7163.MCT-17-0349

[9] Alakus H , Babicky ML , Ghosh P , et al. Genome‐wide mutational landscape of mucinous carcinomatosis peritonei of appendiceal origin. Genome Med. 2014;6(5):43. 24944587 10.1186/gm559 PMC4062050

[10] Fecteau RE , Lutterbaugh J , Markowitz SD , Willis J , Guda K . GNAS mutations identify a set of right‐sided, RAS mutant, villous colon cancers. PLoS ONE. 2014;9(1):e87966. 24498230 10.1371/journal.pone.0087966 PMC3907576

[11] Hugen N , Simons M , Halilovic A , et al. The molecular background of mucinous carcinoma beyond MUC2. J Pathol Clin Res. 2015;1(1):3‐17. 27499889 10.1002/cjp2.1 PMC4858120

[12] Liu X , Mody K , de Abreu FB , et al. Molecular profiling of appendiceal epithelial tumors using massively parallel sequencing to identify somatic mutations. Clin Chem. 2014;60(7):1004‐1011. 24821835 10.1373/clinchem.2014.225565

[13] Nishikawa G , Sekine S , Ogawa R , et al. Frequent GNAS mutations in low‐grade appendiceal mucinous neoplasms. Br J Cancer. 2013;108(4):951‐958. 23403822 10.1038/bjc.2013.47 PMC3590682

[14] Noguchi R , Yano H , Gohda Y , et al. Molecular profiles of high‐grade and low‐grade pseudomyxoma peritonei. Cancer Med. 2015;4(12):1809‐1816. 26475379 10.1002/cam4.542 PMC5123786

[15] Nummela P , Saarinen L , Thiel A , et al. Genomic profile of pseudomyxoma peritonei analyzed using next‐generation sequencing and immunohistochemistry. Int J Cancer. 2015;136(5):E282‐289. 25274248 10.1002/ijc.29245

[16] Pietrantonio F , Perrone F , Mennitto A , et al. Toward the molecular dissection of peritoneal pseudomyxoma. Ann Oncol. 2016;27(11):2097‐2103. 27502722 10.1093/annonc/mdw314

[17] Sekine S , Ogawa R , Oshiro T , et al. Frequent lack of GNAS mutations in colorectal adenocarcinoma associated with GNAS‐mutated villous adenoma. Genes Chromosomes Cancer. 2014;53(4):366‐372. 24470207 10.1002/gcc.22147

[18] Singhi AD , Davison JM , Choudry HA , et al. GNAS is frequently mutated in both low‐grade and high‐grade disseminated appendiceal mucinous neoplasms but does not affect survival. Hum Pathol. 2014;45(8):1737‐1743. 24925222 10.1016/j.humpath.2014.04.018

[19] Cancer Genome Atlas N . Comprehensive molecular characterization of human colon and rectal cancer. Nature. 2012;487(7407):330‐337. 22810696 10.1038/nature11252 PMC3401966

[20] Hasnain SZ , Lourie R , Das I , Chen AC , McGuckin MA . The interplay between endoplasmic reticulum stress and inflammation. Immunol Cell Biol. 2012;90(3):260‐270. 22249202 10.1038/icb.2011.112 PMC7165805

[21] Li X , Zhang K , Li Z . Unfolded protein response in cancer: the physician's perspective. J Hematol Oncol. 2011;4:8. 21345215 10.1186/1756-8722-4-8 PMC3060154

[22] McGuckin MA , Eri RD , Das I , Lourie R , Florin TH . ER stress and the unfolded protein response in intestinal inflammation. Am J Physiol Gastrointest Liver Physiol. 2010;298(6):G820‐832. 20338921 10.1152/ajpgi.00063.2010

[23] Schonthal AH . Endoplasmic reticulum stress: its role in disease and novel prospects for therapy. Scientifica. 2012;2012:857516. 24278747 10.6064/2012/857516 PMC3820435

[24] Darling NJ , Cook SJ . The role of MAPK signalling pathways in the response to endoplasmic reticulum stress. Biochim Biophys Acta. 2014;1843(10):2150‐2163. 24440275 10.1016/j.bbamcr.2014.01.009

[25] Jiang CC , Chen LH , Gillespie S , et al. Inhibition of MEK sensitizes human melanoma cells to endoplasmic reticulum stress‐induced apoptosis. Cancer Res. 2007;67(20):9750‐9761. 17942905 10.1158/0008-5472.CAN-07-2047

[26] Wei X , Yang Z , Rey FE , et al. Fatty acid synthase modulates intestinal barrier function through palmitoylation of mucin 2. Cell Host Microbe. 2012;11(2):140‐152. 22341463 10.1016/j.chom.2011.12.006 PMC3285413

[27] Dilly AK , Honick BD , Lee YJ , et al. Targeting G‐protein coupled receptor‐related signaling pathway in a murine xenograft model of appendiceal pseudomyxoma peritonei. Oncotarget. 2017;8(63):106888‐106900. 29290997 10.18632/oncotarget.22455 PMC5739782

[28] Dilly AK , Lee YJ , Zeh HJ , Guo ZS , Bartlett DL , Choudry HA . Targeting hypoxia‐mediated mucin 2 production as a therapeutic strategy for mucinous tumors. Transl Res. 2016;169(19–30):e11. 10.1016/j.trsl.2015.10.006 26589109

[29] Dilly AK , Song X , Zeh HJ , et al. Mitogen‐activated protein kinase inhibition reduces mucin 2 production and mucinous tumor growth. Transl Res. 2015;166(4):344‐354. 25890193 10.1016/j.trsl.2015.03.004

[30] Mahe MM , Sundaram N , Watson CL , Shroyer NF , Helmrath MA . Establishment of human epithelial enteroids and colonoids from whole tissue and biopsy. J Vis Exp. 2015(97):52483. 10.3791/52483 PMC4401205 25866936

[31] Lebre MC , Kalinski P , Das PK , Everts V . Inhibition of contact sensitizer‐induced migration of human Langerhans cells by matrix metalloproteinase inhibitors. Arch Dermatol Res. 1999;291(7–8):447‐452. 10482016 10.1007/s004030050436

[32] Wan J , Roth AF , Bailey AO , Davis NG . Palmitoylated proteins: purification and identification. Nat Protoc. 2007;2(7):1573‐1584. 17585299 10.1038/nprot.2007.225

[33] Charan J , Kantharia ND . How to calculate sample size in animal studies? J Pharmacol Pharmacother. 2013;4(4):303‐306. 24250214 10.4103/0976-500X.119726 PMC3826013

[34] Kerstjens M , Pinhancos SS , Castro PG , et al. Trametinib inhibits RAS‐mutant MLL‐rearranged acute lymphoblastic leukemia at specific niche sites and reduces ERK phosphorylation in vivo. Haematologica. 2018;103(4):e147‐e150. 29419436 10.3324/haematol.2017.174060 PMC5865421

[35] Kuracha MR , Thomas P , Loggie BW , Govindarajan V . Bilateral blockade of MEK‐ and PI3K‐mediated pathways downstream of mutant KRAS as a treatment approach for peritoneal mucinous malignancies. PLoS ONE. 2017;12(6):e0179510. 28640835 10.1371/journal.pone.0179510 PMC5480880

[36] LoRusso P , Shapiro G , Panday S , et al. A first‐in‐human phase 1b study to evaluate the MEK inhibitor GDC‐0973 combined with the pan‐PI3K inhibitor GDC‐0941, in patients with advanced solid tumors. J Clin Oncol. 2012;30(suppl.):Abstract 2566.

[37] Ramanathan R , Von Hoff D , Eskens F , et al. A phase 1b trial of PI3K inhibitor copanlisib (BAY 80‐6946) combined with the allosteric‐MEK inhibitor refametinib (BAY 86‐9766) in patients with advanced cancer. J Clinic Oncol. 2014;32(15_suppl):2588‐2588.

[38] Bedard PL , Tabernero J , Janku F , et al. A phase Ib dose‐escalation study of the oral pan‐PI3K inhibitor buparlisib (BKM120) in combination with the oral MEK1/2 inhibitor trametinib (GSK1120212) in patients with selected advanced solid tumors. Clin Cancer Res. 2015;21(4):730‐738. 25500057 10.1158/1078-0432.CCR-14-1814

[39] Juric D , Soria J‐C , Sharma S , et al. A phase 1b dose‐escalation study of BYL719 plus binimetinib (MEK162) in patients with selected advanced solid tumors. J Clin Oncol. 2014;32(15_suppl):9051‐9051.

[40] Bettington M , Walker N , Clouston A , Brown I , Leggett B , Whitehall V . The serrated pathway to colorectal carcinoma: current concepts and challenges. Histopathology. 2013;62(3):367‐386. 23339363 10.1111/his.12055

[41] Khan M , Loree JM , Advani SM , et al. Prognostic implications of mucinous differentiation in metastatic colorectal carcinoma can be explained by distinct molecular and clinicopathologic characteristics. Clin Colorectal Cancer. 2018;17(4):e699‐e709. 30205948 10.1016/j.clcc.2018.07.005 PMC6588353

[42] Tanaka H , Deng G , Matsuzaki K , et al. BRAF mutation, CpG island methylator phenotype and microsatellite instability occur more frequently and concordantly in mucinous than non‐mucinous colorectal cancer. Int J Cancer. 2006;118(11):2765‐2771. 16381005 10.1002/ijc.21701

[43] Karagkounis G , Torbenson MS , Daniel HD , et al. Incidence and prognostic impact of KRAS and BRAF mutation in patients undergoing liver surgery for colorectal metastases. Cancer. 2013;119(23):4137‐4144. 24104864 10.1002/cncr.28347 PMC3967132

[44] Kufe DW . Mucins in cancer: function, prognosis and therapy. Nat Rev Cancer. 2009;9(12):874‐885. 19935676 10.1038/nrc2761 PMC2951677

[45] Choudry HA , O'Malley ME , Guo ZS , Zeh HJ , Bartlett DL . Mucin as a therapeutic target in pseudomyxoma peritonei. J Surg Oncol. 2012;106(7):911‐917. 22585683 10.1002/jso.23146

[46] Van Seuningen I , Pigny P , Perrais M , Porchet N , Aubert JP . Transcriptional regulation of the 11p15 mucin genes. Towards new biological tools in human therapy, in inflammatory diseases and cancer? Front Biosci. 2001;6:D1216‐1234. 11578973 10.2741/seuning

