# Supplementary figures and images for: Rational application of targeted therapeutics in mucinous colon/appendix cancers with positive predictive factors
Source: Cancer Med. 2020 Jan 20;9(5):1753–67. doi: 10.1002/cam4.2847 (PMC7050077; doi:10.1002/cam4.2847)

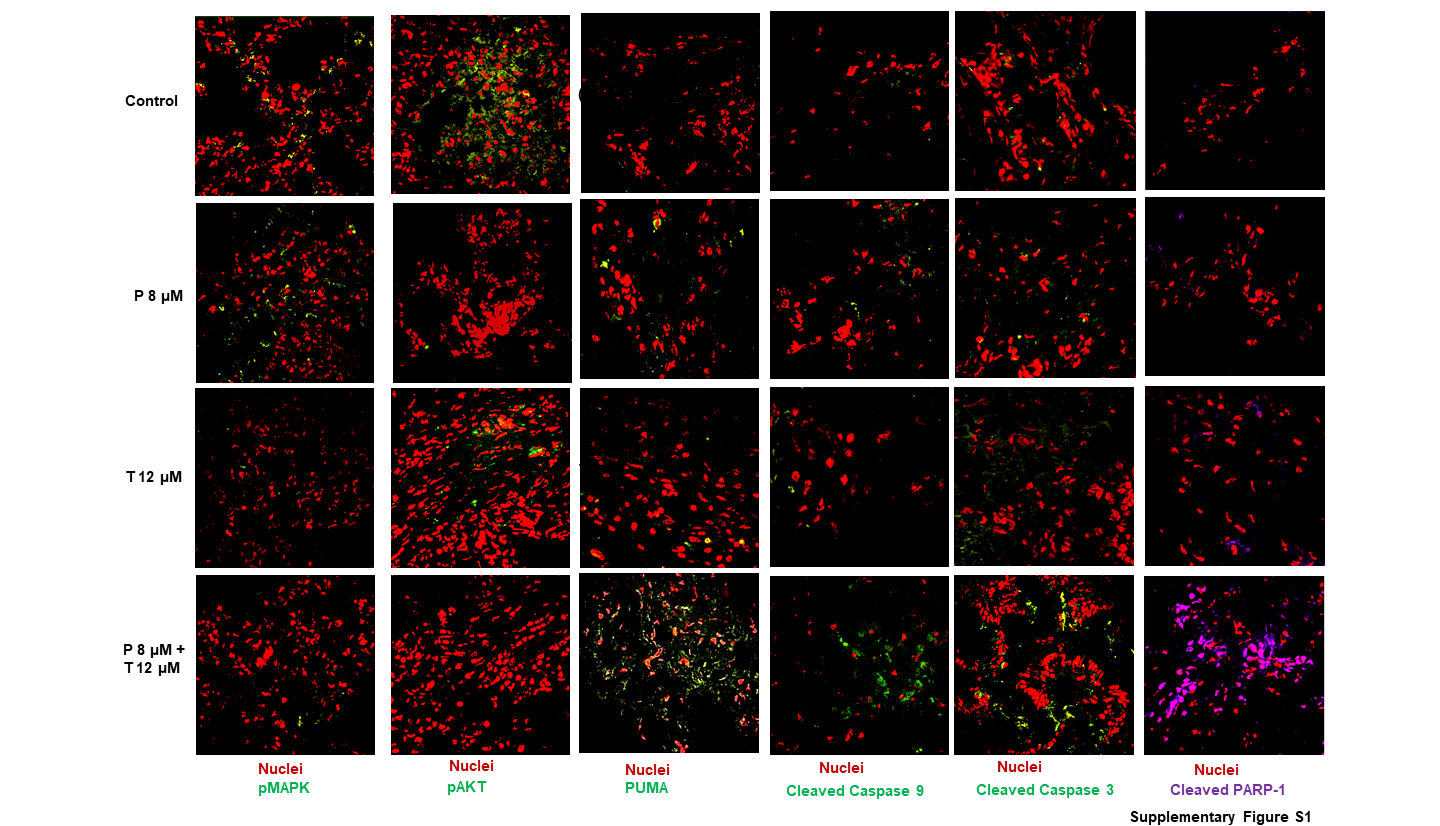

Supplement: Supplementary file 1 [file CAM4-9-1753-s001.tif]

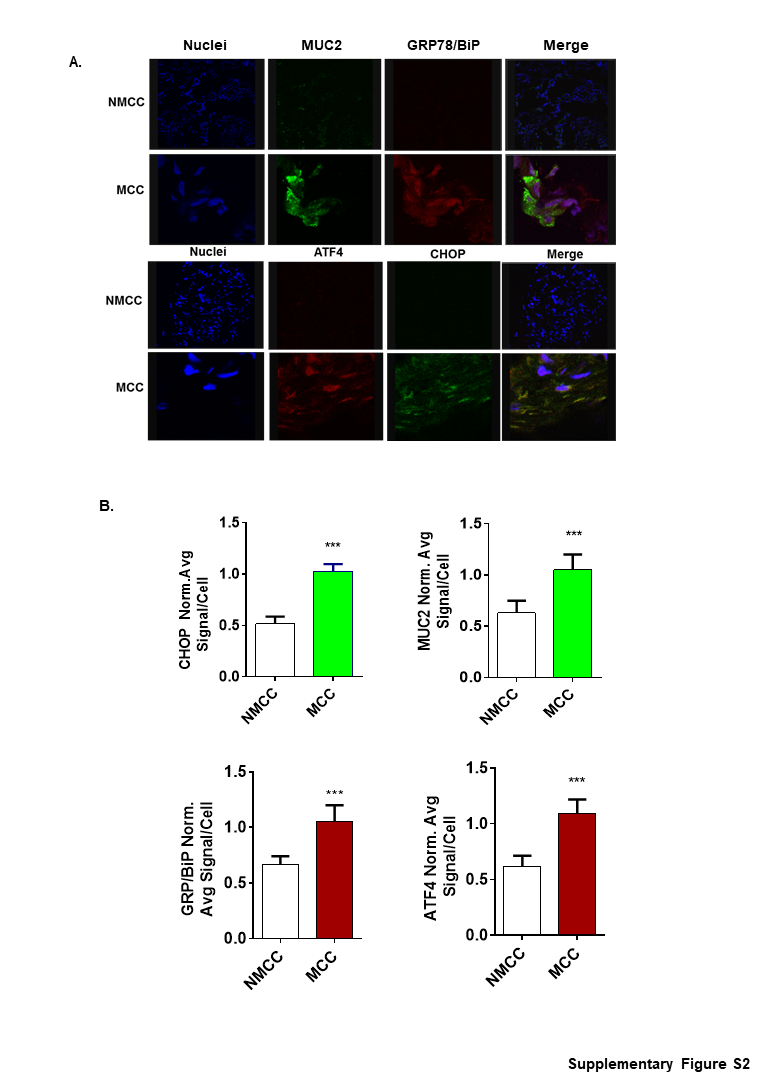

Supplement: Supplementary file 2 [file CAM4-9-1753-s002.tif]

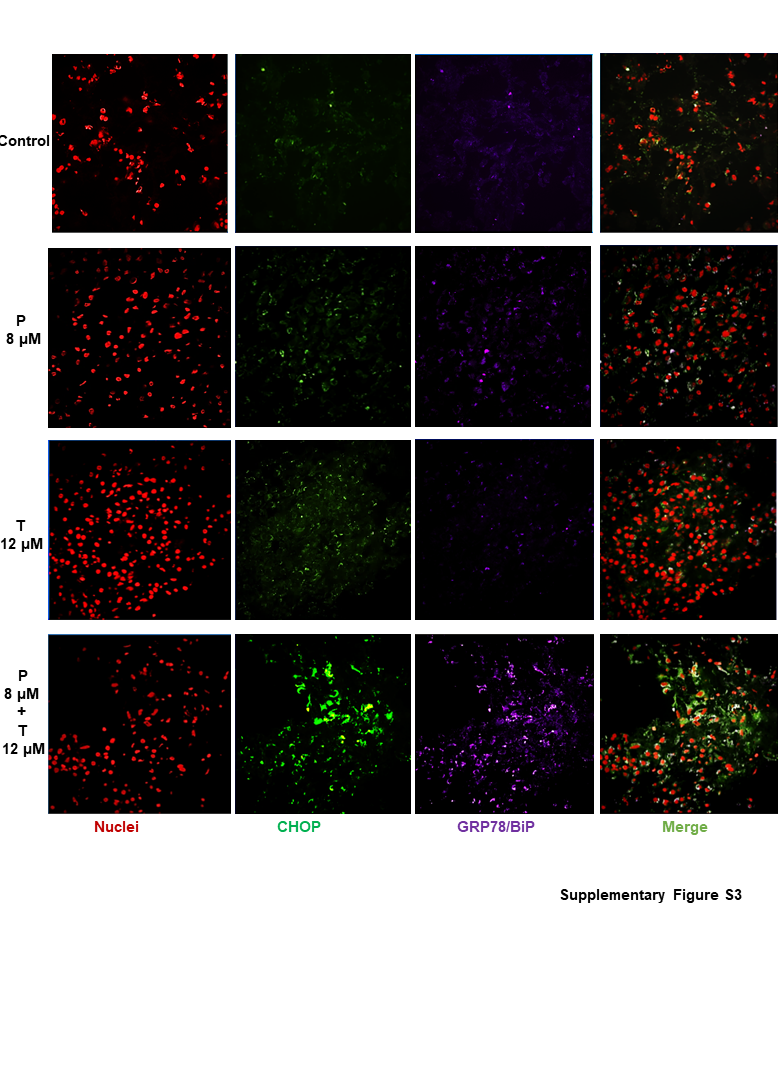

Supplement: Supplementary file 3 [file CAM4-9-1753-s003.tif]
